# New‐user and prevalent‐user designs and the definition of study time origin in pharmacoepidemiology: A review of reporting practices

**DOI:** 10.1002/pds.5258

**Published:** 2021-05-10

**Authors:** Kim Luijken, Judith J. Spekreijse, Maarten van Smeden, Helga Gardarsdottir, Rolf H. H. Groenwold

**Affiliations:** ^1^ Department of Clinical Epidemiology Leiden University Medical Center Leiden The Netherlands; ^2^ Department of Paediatrics Diakonessenhuis Utrecht The Netherlands; ^3^ Julius Center for Health Sciences and Primary Care, University Medical Center Utrecht Utrecht University Utrecht The Netherlands; ^4^ Division of Pharmacoepidemiology and Clinical Pharmacology, Utrecht Institute for Pharmaceutical Sciences Utrecht University Utrecht The Netherlands; ^5^ Department of Clinical Pharmacy, Division Laboratories, Pharmacy and Biomedical Genetics, University Medical Center Utrecht Utrecht University Utrecht The Netherlands; ^6^ Faculty of Pharmaceutical Sciences University of Iceland Reykjavik Iceland; ^7^ Department of Biomedical Data Sciences Leiden University Medical Center Leiden The Netherlands

**Keywords:** causal inference, new‐user design, pharmacoepidemiology, prevalent‐user design

## Abstract

**Background:**

Guidance reports for observational comparative effectiveness and drug safety research recommend implementing a new‐user design whenever possible, since it reduces the risk of selection bias in exposure effect estimation compared to a prevalent‐user design. The uptake of this guidance has not been studied extensively.

**Methods:**

We reviewed 89 observational effectiveness and safety cohort studies published in six pharmacoepidemiological journals in 2018 and 2019. We developed an extraction tool to assess how frequently new‐user and prevalent‐user designs were reported to be implemented. For studies that implemented a new‐user design in both treatment arms, we extracted information about the extent to which the moment of meeting eligibility criteria, treatment initiation, and start of follow‐up were reported to be aligned.

**Results:**

Of the 89 studies included, 40% reported implementing a new‐user design for both the study exposure arm and the comparator arm, while 13% reported implementing a prevalent‐user design in both arms. The moment of meeting eligibility criteria, treatment initiation, and start of follow‐up were reported to be aligned in both treatment arms in 53% of studies that reported implementing a new‐user design. We provided examples of studies that minimized the risk of introducing bias due to unclear definition of time origin in unexposed participants, immortal time, or a time lag.

**Conclusions:**

Almost half of the included studies reported implementing a new‐user design. Implications of misalignment of study design origin were difficult to assess because it would require explicit reporting of the target estimand in original studies. We recommend that the choice for a particular study time origin is explicitly motivated to enable assessment of validity of the study.

**Key Points**

- Literature about recent pharmacoepidemiologic effectiveness and safety cohort studies of drug‐outcome associations was reviewed to assess the reporting of implementation of and rationale for using new‐user and prevalent‐user designs.
- Almost half of the included studies reported to follow the recommendation to implement a new‐user design. Rationales for implementing a prevalent‐user design were scarcely reported.
- The study time origin and allocation of follow‐up time influence the extent to which the available data can provide a meaningful estimate of the causal effect of interest. We recommend that the choice for a particular study time origin is explicitly motivated to enable assessment of validity of the study.

## 1 INTRODUCTION

Guidance reports for comparative effectiveness and safety research of pharmacological treatments recommend the new‐user design,^1^, ^2^, ^3^, ^4^ in which follow‐up time generally starts with the first prescription or dispensing of the drug(s) of interest.^5^ In contrast, in the prevalent‐user design both current (prevalent) and new users of a drug are included. The new‐user design enforces appropriate temporal ordering of measurements of confounders, treatment, and outcome, protecting the researcher against accidental adjustment for variables affected by treatment and against finding associations that are based on reversed causation^1^, ^2^, ^3^, ^4^, ^5^, ^6^, ^7^, ^8^ However, the start of a treatment can be difficult to capture (especially in case of intermittently used treatments) and exclusion of prevalent users may reduce follow‐up time or sample size^5^, ^7^, ^8^, ^9^, ^10^ It is unclear how often and for which reasons researchers deviated from the guidance to implement a new‐user design.

To assess the uptake of new‐user design guidance, it is important to go beyond the distinction of including new or prevalent users. Many time‐related biases can be prevented by choosing a study time origin (or study baseline) such that it establishes alignment of the moment of meeting eligibility criteria, treatment initiation, and start of follow‐up.^6^, ^11^, ^12^, ^13^ Previous studies investigated how often pharmacoepidemiological studies deviated from the recommendation to implement a new‐user design,^14^, ^15^, ^16^ however, the implementation of new‐user designs in terms of alignment of eligibility, treatment initiation, and start of follow‐up has not been studied yet.

In the current study, we reviewed the literature about contemporary observational effectiveness and safety cohort studies. We assessed how frequently new‐user and prevalent‐user designs were reported to be implemented in studies published in high‐ranked pharmacoepidemiologic journals. For studies implementing a new‐user design, we evaluated to what extent eligibility, treatment initiation, and start of follow‐up were reported to be aligned.

## 2 METHODS

We systematically assessed the reporting practices in observational studies of treatment effects regarding the definition of the study time origin and inclusion of new versus prevalent users of treatment. A protocol of this study is available on Open Science Framework.^17^ Based on recommendations by the editor and reviewers, we deviated from this protocol. Specifically, while we scored the items of the extraction tool for all included articles, we discuss the results on alignment in study design origin for new‐user designs only, as will be explained below. This review followed the Preferred Reporting Items for Systematic Reviews and Meta‐Analyses (PRISMA) guidelines,^18^ where applicable.

### 2.1 Journal selection and included type of studies

We aimed to review the reporting of approximately 100 articles published before the 1st of July 2019 in journals publishing pharmacoepidemiologic studies of drug‐outcome associations. Six pharmacoepidemiological journals were included: Annals of Pharmacotherapy, British Journal of Clinical Pharmacology, Drug Safety, European Journal of Clinical Pharmacology, Pharmacotherapy, and Pharmacoepidemiology and Drug Safety. These state‐of‐the art pharmacoepidemiological journals were selected because reporting on study design implementation was expected to be relatively complete. We performed a PubMed search on February 3rd 2020 (see protocol^17^ and Supplementary materials for search string) which returned 2,457 records. Study inclusion criteria were: study described original pharmacoepidemiologic research into the relation between drug exposure and a clinical outcome; data were collected for research purposes or obtained from routinely collected health data; the data were gathered according to a cohort study design, since the definition of new versus prevalent users is not as straightforward in other designs, such as a cross‐sectional, case‐crossover or case–control design. Exclusion criteria were: pharmacokinetic‐pharmacodynamic studies; cost‐effectiveness studies; data on treatment exposure were collected through self‐report. We also excluded studies of vaccination, antibiotic treatment of a single treatment episode (up to 10 days), chemotherapy, or intravenous drugs, because for these kinds of interventions new‐user designs are more natural. KL screened the title and abstract of all studies that result from the searches and included relevant articles based on the eligibility criteria. We applied a quota sampling strategy^19^ and continued screening articles until we reached the most recent 100 articles published before July 1st, 2019.

### 2.2 Extraction of study characteristics and evaluation of reporting quality

Articles were scored on a set of items derived from guideline recommendations about elements that should be reported in protocols^20^, ^21^ or articles^4^, ^22^ of effectiveness and safety research using large observational databases, as well as methodological articles that discuss the study time origin in observational studies of causal effects.^6^, ^11^ The main focus was on the distinction between new‐user and prevalent‐user designs and the alignment of moment of meeting eligibility criteria, moment of treatment initiation, and start of follow‐up in new‐user designs. The rationale for alignment of meeting eligibility, treatment initiation, and start of follow‐up is described in the Data S1, as well as possible consequences of misalignment. The established scoring tool was pilot tested on six randomly chosen included studies by KL and JS and further adjusted (all items can be found in Tables 2 and 3).

An incident user can more generally be defined as a new user of any treatment decision, that is, initiating a treatment, but also switching to a different treatment or a change of dose. This understanding of the new‐user design was introduced by Brookhart,^23^ expanded to prevalent new‐users of treatment by Suissa,^24^ and was followed during scoring of articles. For the item that scored reporting of whether the comparator exposure arm implemented a new‐user or prevalent‐user design, we decided to score nonusers of treatment as prevalent users. Whereas nonuse is not associated with the biases typically associated with prevalent users (eg, adjusting for intermediates, depletion of susceptibles), definition of study time origin in studies with a nonuser comparator arm is complicated because the choice of the time origin since which the (cumulative) probability of an event of interest can occur in the specified population may not be as straightforward for nonusers of treatment. Consequently, it is more challenging to assess whether the study exposure arm and comparator arm can be assumed to be comparable conditional on measured confounders (ie, whether there is conditional exchangeability).

Information was gathered on general characteristics of the included studies; funding source, type of data source, patient domain, sample size, and length of enrollment window. Funding source was defined as “private” when the article stated the study was funded by a pharmaceutical company or when any of the authors was affiliated with a pharmaceutical company and defined as “public” otherwise. Data sources were classified into hospital data, dispensings, prescriptions, or claims. Patient domain was grouped into medical specialties based on the target population that was mentioned in the article objective. When the target population did not match a single medical specialty, information on the type of treatment and study outcome was used to identify the medical specialty.

Articles were reviewed independently by KL and JS, results were discussed between the two reviewers and in case of disagreement a third reviewer (RG) was consulted. When multiple effectiveness or safety analyses were described in a single article, only the first‐reported analysis was scored. When subgroup analyses were performed in the included studies, only the main analysis was scored. When methods were discussed in an online protocol or described in a different article, we reviewed the referred material.

### 2.3 Data synthesis

Rater agreement was computed using the unweighted Cohen's kappa for nominal variables and two coders.^25^ Cohen's kappa ranges from −1 (perfect disagreement) to 1 (perfect agreement). Reporting of items was presented as percentages of total number of included studies and 95% confidence intervals (CIs) were computed using the normal approximation.

## 3 RESULTS

**FIGURE 1:**
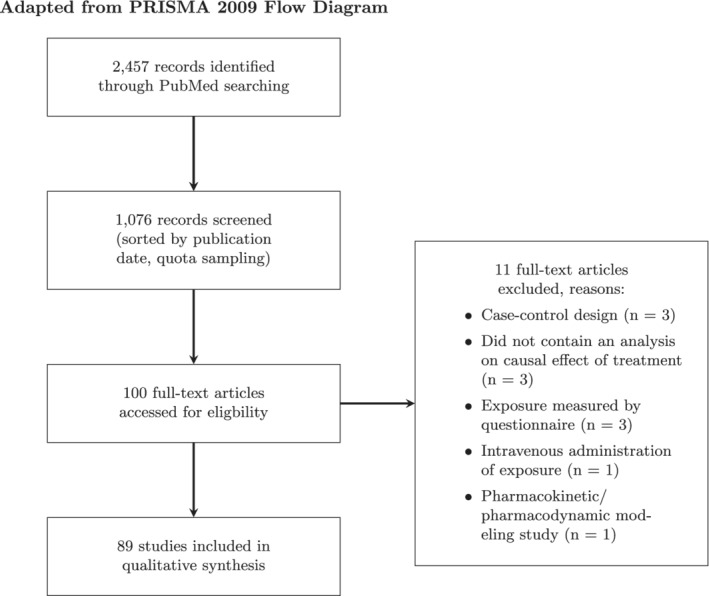
The screening and inclusion of eligible articles

**FIGURE 2:**
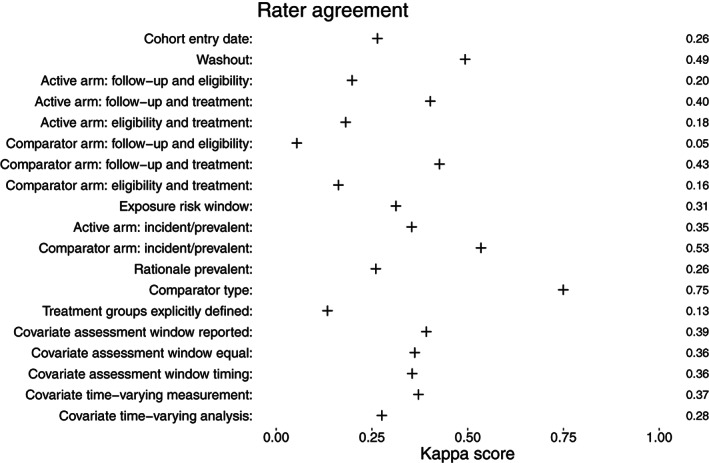
Agreement between raters, measured by Cohen's kappa (unweighted)

After screening the full texts of the 100 articles included during abstract and title screening, 89 studies remained based on the eligibility criteria (see Figure 1). The characteristics of the 89 included studies are summarized in Table 1. The most common patient domains considered were cardiology (17 %), neurology (11%) and primary care (10%). The median sample size was 7,011 (range 14‐3,351,674). In 10% of studies (n = 9), a sample size calculation was reported. The length of follow‐up ranged from 1 hour follow‐up in one study to a median follow‐up of 13.6 years in another study. Rater agreement is presented in Figure 2. Item kappas indicated that agreement between raters was low (range 0.05‐0.75), which was mostly due to ambiguous reporting of the extracted information. Despite the low rater agreement of the initial scores, the presented results have a meaningful interpretation since consensus was reached for all scores with initial disagreement.

**TABLE 1:** Characteristics of the 89 included studies.

Item	Item options	Number of studies (proportion)
Journal	Annals of Pharmacotherapy	16 (0.18)
	British Journal of Clinical Pharmacology	12 (0.13)
	Drug Safety	11 (0.12)
	European Journal of Clinical Pharmacology	8 (0.09)
	Pharmacoepidemiology and Drug Safety	27 (0.30)
	Pharmacotherapy	15 (0.17)
Continent	Africa	1 (0.01)
	Asia	16 (0.18)
	Europe	30 (0.34)
	North America	37 (0.42)
	Oceania	2 (0.02)
	Multiple	1 (0.01)
	Not reported	2 (0.02)
Year of publication	2018	56 (0.63)
	2019	33 (0.27)
Funding	Nonpharmaceutical	83 (0.93)
	Pharmaceutical	6 (0.07)
Data source type	Claims	32 (0.36)
	Dispensing	19 (0.21)
	Hospital data	26 (0.29)
	Prescription	11 (0.12)
	Dispensing and prescription	1 (0.01)
Domain	Cardiology	15 (0.17)
	Neurology	10 (0.11)
	Primary care	9 (0.10)
	Infectious disease	6 (0.07)
	Nephrology	6 (0.07)
	Other	43 (0.48)
Sample size	< 500	23 (0.26)
	500–50 000	44 (0.49)
	> 50 000	22 (0.25)
Sample size calculation	No	80 (0.90)
	Yes	9 (0.10)
If sample size calculation	No	1 (0.11)
Reported, size reached?	Yes	7 (0.78)
	Unclear	1 (0.11)
Cohort entry^10^	Event‐based	22 (0.25)
	Exposure‐based	28 (0.31)
	Multiple event‐based	33 (0.37)
	Time‐based	6 (0.07)
Study entry level^3^ ^,item C2^	Episode	6 (0.07)
	Person	83 (0.93)

### 3.1 New‐user and prevalent‐user designs

**FIGURE 3:**
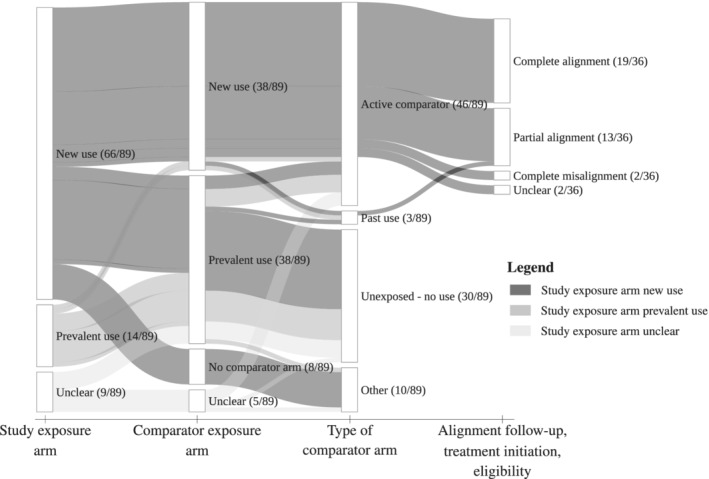
Frequency of reporting of implementation of new‐user and prevalent‐user design and type of comparator across the 89 included studies. For studies that reported implementing a new‐user design, alignment of eligibility, treatment initiation and follow‐up was scored “completely aligned” when all three elements were reported to be aligned in both the active and comparator exposure arm; “completely misaligned” when none of the elements were reported to be aligned in both the active and comparator exposure arm; “unclear” when all three elements were unclear in both the active and comparator exposure arm; “partial alignment” otherwise

An overview of item scores is given in Table 2. Forty percent of studies (95% CI 30% ‐51%, n = 36) reported implementing a new‐user design for both the study exposure arm and the comparator exposure arm, while 13% (7%‐22%, n = 12) reported implementing a prevalent‐user design for both treatment arms (Figure 3). In 58% (42%‐74%, n = 21) of studies with a new‐user design for both treatment arms a washout for exposure was reported. For 6% of studies (1%‐10%, n = 5) it was unclear whether a new‐user or a prevalent‐user design was implemented. When a prevalent‐user design was reported to be implemented, three studies provided a rationale for including prevalent users. The motivation to include prevalent users concerned biological plausibility of a cumulative effect on outcome risk^26^, ^27^, ^28^ .

**TABLE 2:** Summary of reporting of information extracted from 89 reviewed articles.

Item	Item options	Number of studies	Proportion (95% confidence interval)
Study exposure arm			
New/prevalent users	New users	66	0.74 (0.65; 0.83)
	Prevalent users	14	0.16 (0.08; 0.23)
	Unclear	9	0.10 (0.04; 0.16)
Comparator exposure arm			
Comparator type	Active comparator	46	0.52 (0.41; 0.62)
	Unexposed – no use	30	0.34 (0.24; 0.44)
	Unexposed – past use	3	0.03 (0.00; 0.07)
	Combination	1	0.01 (0.00; 0.03)
	Other	6	0.07 (0.02; 0.12)
	No comparator specified	3	0.03 (0.00; 0.07)
New/prevalent users	New users	38	0.43 (0.32; 0.53)
	Prevalent users	38	0.43 (0.32; 0.53)
	Unclear	5	0.06 (0.01; 0.10)
	No comparator or symmetry design	8	0.09 (0.03; 0.15)
General design features			
Treatment groups explicitly defined	Yes	84	0.94 (0.90; 0.99)
	No	5	0.06 (0.01; 0.10)
Cohort entry date reported	Yes	71	0.80 (0.71; 0.88)
	No	18	0.20 (0.12; 0.29)
Washout reported	Yes	37	0.42 (0.31; 0.52)
	No	52	0.58 (0.48; 0.69)
Exposure risk window reported	Yes	74	0.83 (0.75; 0.91)
	No	15	0.17 (0.09; 0.25)
Covariate assessment			
Covariate assessment window reported	Yes	45	0.51 (0.40; 0.61)
	No	38	0.43 (0.32; 0.53)
	Symmetry design or self‐controlled	6	0.07 (0.02; 0.12)
If covariate assessment window was reported (n = 45), was the covariate assessment window equal for all covariates	Yes	20	0.44 (0.30; 0.59)
	No	24	0.53 (0.39; 0.68)
	Not reported	1	0.02 (0.00; 0.07)
If covariate assessment window was reported (n = 45), was the covariate assessment window before initiation of treatment	Yes	27	0.60 (0.46; 0.74)
	No	13	0.27 (0.14; 0.40)
	Not reported	5	0.11 (0.02; 0.20)
If exposure was time‐varying (n = 18), were covariates measured time‐varying	Yes	9	0.50 (0.27; 0.73)
	No	6	0.33 (0.12; 0.55)
	Not reported	3	0.16 (0.00; 0.34)
If covariates were measured time‐varying (n = 12), was this incorporated in analysis	Yes	7	0.58 (0.30; 0.86)
	No	1	0.08 (0.00; 0.24)
	Not reported	4	0.33 (0.07; 0.60)

### 3.2 Alignment in new‐user designs

In the 36 studies that reported implementing a new‐user design in both treatment arms, moment of meeting eligibility criteria, treatment initiation, and start of follow‐up were reported to be aligned in both treatment arms in 53% of studies (36%‐69%, n = 19). Moment of meeting eligibility criteria, start of treatment, and start of follow‐up were reported to be misaligned in both treatment arms in 6% of studies (0%‐13%, n = 2) and alignment was unclear in 6% of studies (0%‐13%, n = 2) (Figure 3). In the remaining studies (n = 13), at least one of the six alignment items was misaligned or unclear (see Table 3 for the alignment items).

**TABLE 3:** Summary of reporting of alignment of start of follow‐up, meeting eligibility criteria and treatment initiation extracted from 36 articles that implemented a new‐user design in both treatment arms.

Item	Item options	Number of studies	Proportion (95% confidence interval)
Study exposure arm			
Alignment follow‐up—eligibility	Yes	24	0.67 (0.51; 0.82)
	No	9	0.25 (0.11; 0.39)
	Unclear	3	0.08 (0.00; 0.17)
Alignment follow‐up—treatment	Yes	26	0.72 (0.58; 0.87)
	No	3	0.08 (0.00; 0.17)
	Unclear	7	0.19 (0.07; 0.32)
Alignment eligibility—treatment	Yes	22	0.61 (0.45; 0.77)
	No	9	0.25 (0.11; 0.39)
	Unclear	5	0.14 (0.03; 0.25)
Comparator exposure arm			
Alignment follow‐up—eligibility	Yes	21	0.58 (0.42; 0.74)
	No	11	0.31 (0.16; 0.46)
	Unclear	4	0.11 (0.01; 0.21)
Alignment follow‐up—treatment	Yes	25	0.69 (0.54; 0.84)
	No	5	0.14 (0.03; 0.25)
	Unclear	6	0.17 (0.04; 0.29)
Alignment eligibility—treatment	Yes	20	0.56 (0.39; 0.72)
	No	12	0.33 (0.18; 0.49)
	Unclear	4	0.11 (0.01; 0.21)

Implications of misalignment of eligibility, treatment initiation, and start of follow‐up can only be assessed relative to the specified causal contrast of interest, that is, the target causal effect or so‐called estimand. Initially, the protocol of this study contained an item to extract whether the target estimand was reported, but we adjusted this during the pilot phase of our extraction tool when we discovered that no study explicitly reported a target estimand (see protocol revision^17^ from version 2 to version 3). Based on recommendations by the editor and reviewers, we scored whether an explicit description of the target estimand was provided in the 36 new‐user active‐comparator studies. Twenty‐two percent of studies (9%‐36%, n = 8) provided an explicit definition of the target estimand. In studies that did not explicitly report the target estimand, it was often unclear which treatment strategies were compared and which treatment decision could be informed based on evidence from the conducted study.

### 3.3 Examples of good practice

Using examples from the 89 included studies, the next section illustrates how study designs that deviate from an archetypical pharmacoepidemiological active‐comparator new‐user design could still provide estimates of the target treatment effect with a meaningful interpretation. We did not find any examples with a meaningfully defined study time origin among studies that contained a prevalent‐user active‐comparator arm.

#### 3.3.1 Study design with nonuser comparator arm

Korol and colleagues investigated whether initiation of spironolactone affected the risk of new onset diabetes in older patients with heart failure compared to not initiating spironolactone.^29^ The patient cohort was defined by day of discharge of the first hospitalization for heart failure. The follow‐up was started at the date of first dispensed prescription of spironolactone for the study exposure arm. The start of follow‐up for unexposed comparator patients was inherited from the cohort entry date of the comparator and set to the time since hospital discharge from their matched comparator to establish a meaningful study time origin for nonusers, given additional implementations to meet assumptions such as measuring sufficient confounders to invoke the exchangeability assumption (Table 4). Note that when an event‐based cohort is established, resetting the start of follow‐up at the moment of treatment initiation or comparable duration since diagnosis is essential to prevent introduction of immortal time bias.^11^

**TABLE 4:** Examples of design solutions for study time origin.

Research question	Designed time origin	Study time origin
Does initiation of spironolactone affect the risk of new‐onset diabetes in older patients with heart failure compared to nonuse of spironolactone?^29^	The patient cohort was defined by day of discharge of the first hospitalization for heart failure. For the study exposure arm, the follow‐up was started at the date of first out‐of‐hospital dispensed prescription of spironolactone. The date of start of follow‐up for unexposed comparator patients was matched to that of exposed patients on the time since hospital discharge axis to establish a meaningful study time origin for nonusers. The authors did not report whether the nonuser cohort was defined based on current exposure information or on future exposure information, that is, whether nonusers could still start spironolactone after their inherited date of start of follow‐up or had to be unexposed during the entire study follow‐up. The latter could result in a comparator cohort that is restricted to individuals who never had an indication for the treatment, which does not necessarily match the causal contrast of interest.^38^	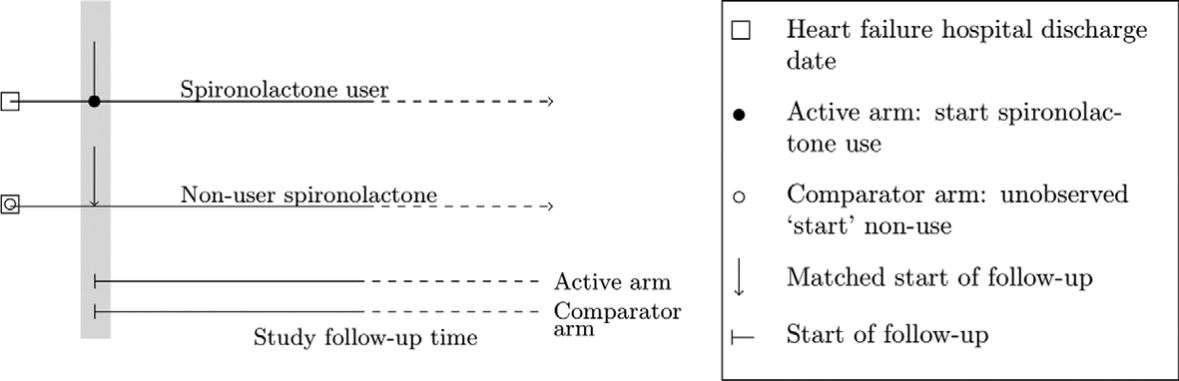
Does initiation of baclofen affect the risk of hospitalization and death compared to initiation of acamprosate in adults with an alcohol use disorder without comorbidities?^30^	The patient cohort was defined by initiation of baclofen/acamprosate. To be eligible, patients had to have received at least a second reimbursement for the same drug within 60 days after the first reimbursement. The start of follow‐up was reset after the second prescription to prevent immortal time bias. The study thus estimates the causal effect of baclofen compared to acamprostate given that everyone filled at least two prescriptions within 60 days and death was prevented in the time until they filled a 2nd prescription.	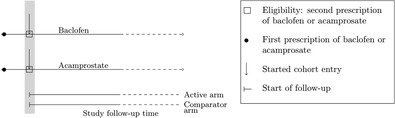
Does switching from epoetin alpha (ESA α) to any other epoetin, compared to not switching, affect the risk of a blood transfusion or developing anemia in chronic kidney disease patients?^31^	The patient cohort was defined by initiation of ESA α. The follow‐up was started at date of switching for the study exposure arm. A matched cohort was created to compare the risk of study outcomes in switchers versus nonswitchers. The start of follow‐up for nonswitchers was matched to duration of ESA α treatment (± 30 days), thereby preventing time‐lag bias (in matching, other covariates were considered as well).	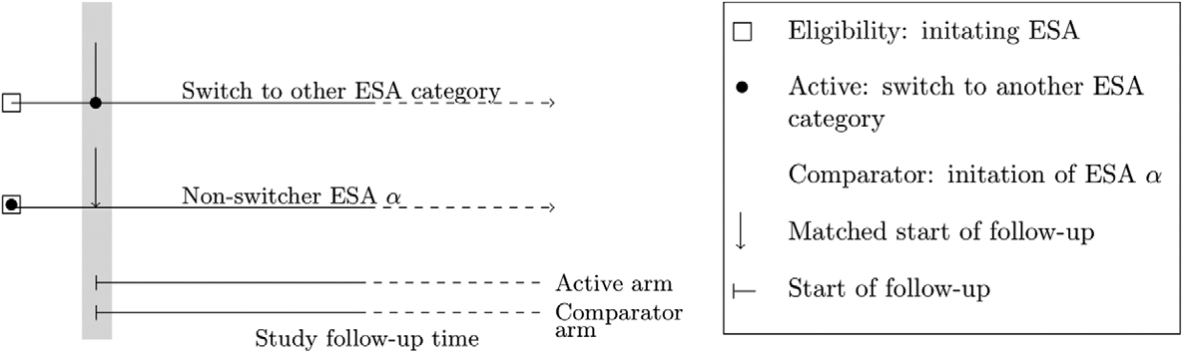

#### 3.3.2 Study design that anticipated immortal time

Chaignot and colleagues studied whether initiation of baclofen affected the risk of hospitalization and death compared to initiation of acamprosate in adults with an alcohol use disorder without comorbidities.^30^ The patient cohort was defined by initiation of baclofen/acamprosate. To be eligible, patients had to receive at least two reimbursements for the same drug within 60 days after the first reimbursement, meaning that for included individuals, hospitalization/death could not have occurred before the second reimbursement was received. The start of follow‐up was reset after the second prescription to prevent immortal time bias (Table 4). Note that the estimand changes by resetting start of follow‐up. The study aims to identify the causal effect of baclofen compared to acamprostate given that everyone filled at least two prescriptions within 60 days and death was prevented during the time until they filled a 2nd prescription. This interpretation is arguably more difficult to translate to clinical practice than a causal effect of initiating baclofen versus initiating acamprostate.

#### 3.3.3 Study design that addressed time lags in start of follow‐up

Belleudi and colleagues investigated whether switching from epoetin alpha (ESA α) to any other epoetin, compared to not switching, affected the risk of a blood transfusion or developing anemia in chronic kidney disease patients.^31^ The patient cohort was defined by initiation of ESA α. The follow‐up was started at date of switching for the study exposure arm. A matched cohort was created to compare the risk of study outcomes in switchers versus nonswitchers. The start of follow‐up for nonswitchers was matched to duration of ESA α treatment (± 30 days), thereby preventing time‐lag bias (Table 4).

## 4 DISCUSSION

In our review of 89 pharmacoepidemiologic cohort studies of drug‐outcome associations, 40% reported implementing a new‐user design for both the study exposure arm and the comparator exposure arm, while 13% reported implementing a prevalent‐user design in both arms, and three studies provided a rationale for including prevalent users. In studies that reported implementing a new‐user design, we found there is room for improving alignment of meeting eligibility, treatment initiation, and start of follow‐up, and reporting thereof.

It is not straightforward to understand the implications of misalignment of eligibility, treatment initiation, and start of follow‐up in studies implementing a new‐user design. Misalignment in the operationalization of the time origin in a study design can introduce immortal time bias or time‐lag bias (see Supplementary materials),^3^, ^5^, ^6^, ^7^, ^11^, ^12^, ^32^, ^33^, ^34^ but analytic methods can also help prevent these biases (eg, analyzing treatment as a time‐dependent variable as proposed by Suissa and Azoulay^13^). The validity of the chosen design and analysis is ideally assessed relative to the target estimand. Since target estimands were not often explicitly reported, we were not able to further assess implications of misalignment in the study time origin. It might have been possible to derive the target estimand from information in the methods section in some studies. However, this would not contribute to assessment of the validity of the chosen design and analysis since target and operationalization would then overlap completely because of the reflexive definition of the target. When a target estimand is not reported explicitly, it is unclear which treatment effect the study aims to estimate, making it impossible to assess the impact of misalignment of eligibility, treatment initiation, and start of follow‐up on validity of the study based on what is reported in the article. On the other hand, providing a concise and explicit definition of a target estimand is a challenging task.

Our findings are in line with previous studies that investigated the implementation of the new‐user design in specific patient domains. Yoshida and colleagues reviewed cohort studies investigating the association between use of disease‐modifying antirheumatic drugs and either risk of infections (52 studies) or risk of cancers (15 studies) published between 2005 and 2015.^15^ Forty percent of the studies on infection risk and 27% of the studies on cancer risk implemented a new‐user active‐comparator design, which is similar and lower, respectively, compared to the proportions found in our study, which covered a wider range of research areas. Suissa and Azoulay presented examples of observational studies investigating the association between metformin and cancer that suffered from immortal time bias, time‐lag bias, or time‐window bias.^13^ Time‐window bias can be an issue in case–control analysis and was not addressed here, because we only included cohort studies.

Based on our observations, it is our view that choosing a meaningful time origin is a more fundamental component of the study design than the distinction between new or prevalent users alone. Even when a new‐user design was implemented, some of the articles we reviewed defined the study origin ambiguously. Reporting guidelines, such as RECORD‐PE,^35^ state that study entry criteria and the order in which these criteria were applied to identify the study population should be clearly described. Indicating that a new‐user design was implemented is insufficient to justify validity of a study design and time origin.

Our study had limitations. We focused on study‐design approaches to define a meaningful study time origin. Although data analysis approaches can establish correct allocation of follow‐up time as well,^24^, ^36^ we did not assess them in our review. Misalignment of eligibility, treatment initiation, and start of follow‐up may be appropriate when exposures are evaluated in a time‐dependent manner. Four of the studies that reported implementing a new‐user design studied a time‐dependent exposure, thereby possibly adjusting for any misalignment in the study design. In our review, we assessed how frequently new‐user and prevalent‐user designs were implemented based on the reporting in original articles. It was not always possible to distinguish between lack of reporting and lack of implementation. Our results should therefore be interpreted as a summary of reporting practices on study time origin in six journals. A final limitation is that our search was restricted to a convenience sample of six journals. Arguably, the six selected journals are representing the higher impact, specialist pharmacoepidemiology journals and results may therefore overestimate the quality of reporting of pharmacoepidemiologic studies in general.

The following recommendations for the design of pharmacoepidemiologic studies follow from our work. Reporting the motivation for a chosen study design and providing information on the extent to which moment of meeting eligibility criteria, treatment initiation, and start of follow‐up are aligned improves the transparency and validity of research. We re‐emphasize the importance of the recommendation by Schneeweiss and colleagues^37^ to provide a design diagram, depicting a study's key temporal anchors and their relation to each other. When the target estimand is unknown, it is difficult to assess whether study design and analysis are suitable for providing a meaningful estimate of the treatment effect of interest, in particular for time‐dependent exposures. We recommend to explicitly report the causal contrast that is targeted in a separate statement at the beginning of the methods section. The definition of the target estimand ideally concisely states the target population, the treatment strategies that are compared and how they are contrasted, and the outcome assessment (what and when). The causal contrast then explicates which effect is of interest (eg, an intention‐to‐treat effect, a per‐protocol effect, an effect of treatment duration, or a comparison of treatment regimens).^21^ It should be unambiguous from this statement which future treatment decision can be informed by the study findings. Only when this information is clearly reported, the agreement can be assessed between target estimand and applied study design and data analysis.

## CONFLICT OF INTEREST

No authors report any conflict of interest.

## AUTHOR CONTRIBUTIONS

Kim Luijken, Judith J. Spekreijse, Rolf H. H. Groenwold have made substantial contributions to conception and design, or acquisition of data, or analysis and interpretation of data; Kim Luijken, Judith J. Spekreijse, Maarten van Smeden, Helga Gardarsdottir, Rolf H. H. Groenwold have been involved in drafting the manuscript or revising it critically for important intellectual content; Kim Luijken, Judith J. Spekreijse, Maarten van Smeden, Helga Gardarsdottir, Rolf H. H. Groenwold have given final approval of the version to be published. Kim Luijken, Judith J. Spekreijse, Maarten van Smeden, Helga Gardarsdottir, Rolf H. H. Groenwold agreed to be accountable for all aspects of the work in ensuring that questions related to the accuracy or integrity of any part of the work are appropriately investigated and resolved.

## ETHICS STATEMENT

The authors state that no ethical approval was needed.

The authors state that no patient consent was needed.

## Supporting information


Data S1.

## Appendix. Review references A.

[R1] Adimadhyam S, Schumock GT, Calip GS, Smith Marsh DE, Layden BT, Lee TA. Increased risk of mycotic infections associated with sodium–glucose co‐transporter 2 inhibitors: a prescription sequence symmetry analysis. *British Journal of Clinical Pharmacology* 2019; 85(1): 160–168.

[R2] Ahmadizar F, Ochoa‐Rosales C, Glisic M, Franco OH, Muka T, Stricker BH. Associations of statin use with glycaemic traits and new type 2 diabetes. *British Journal of Clinical Pharmacology* 2019; 85(5): 993–1002.

[R3] Al‐Jazairi AS, Al Alshaykh HA, DiSalvo G, De Vol EB, Alhalees ZY. Assessment of late thromboembolic complications post–fontan procedure in relation to different antithrombotic regimens: 30‐years' follow‐up experience. *Annals of Pharmacotherapy* 2019; 53(8): 786–793.

[R4] Arana A, Margulis AV, McQuay LJ, et al. Variation in cardiovascular risk related to individual antimuscarinic drugs used to treat overactive bladder: a UK cohort study. *Pharmacotherapy: The Journal of Human Pharmacology and Drug Therapy* 2018; 38(6): 628–637.

[R5] Bartlett JW, Renner E, Mouland E, Barnes GD, Kuo L, Ha NB. Clinical safety outcomes in patients with nonvalvular atrial fibrillation on rivaroxaban and diltiazem. *Annals of Pharmacotherapy* 2019; 53(1): 21–27.

[R6] Baumgartner L, Lam K, Lai J, et al. Effectiveness of melatonin for the prevention of intensive care unit delirium. *Pharmacotherapy: The Journal of Human Pharmacology and Drug Therapy* 2019; 39(3): 280–287.

[R7] Belleudi V, Trotta F, Addis A, et al. Effectiveness and safety of switching originator and biosimilar epoetins in patients with chronic kidney disease in a large‐scale Italian cohort study. *Drug Safety* 2019; 42(12): 1437–1447.

[R8] Blin P, Dureau‐Pournin C, Cottin Y, et al. Effectiveness and safety of 110 or 150 mg dabigatran vs. vitamin K antagonists in nonvalvular atrial fibrillation. *British Journal of Clinical Pharmacology* 2019; 85(2): 432–441.

[R9] Bluhmki T, Fietz AK, Stegherr R, et al. Multistate methodology improves risk assessment under time‐varying drug intake—a new view on pregnancy outcomes following coumarin exposure. *Pharmacoepidemiology and Drug Safety* 2019; 28(5): 616–624.

[R10] Boley SP, Stellpflug SJ. A comparison of resource utilization in the management of anticholinergic delirium between physostigmine and nonantidote therapy. *Annals of Pharmacotherapy* 2019; 53(10): 1026–1032.

[R11] Boudreau DM, Chen L, Yu O, Bowles EJA, Chubak J. Risk of second breast cancer events with chronic opioid use in breast cancer survivors. *Pharmacoepidemiology and Drug Safety* 2019; 28(5): 740–753.

[R12] Campbell NL, Lane KA, Gao S, Boustani MA, Unverzagt F. Anticholinergics influence transition from normal cognition to mild cognitive impairment in older adults in primary care. *Pharmacotherapy: The Journal of Human Pharmacology and Drug Therapy* 2018; 38(5): 511–519.

[R13] Capetti AF, Cossu MV, Sterrantino G, et al. Dolutegravir plus rilpivirine as a switch option in cART‐experienced patients: 96‐week data. *Annals of Pharmacotherapy* 2018; 52(8): 740–746.

[R14] Chaignot C, Zureik M, Rey G, Dray‐Spira R, Coste J, Weill A. Risk of hospitalization and death related to baclofen for alcohol use disorders: Comparison with nalmefene, acamprosate, and naltrexone in a cohort study of 165 334 patients between 2009 and 2015 in France. *Pharmacoepidemiology and Drug Safety* 2018; 27(11): 1239–1248.

[R15] Chen J, Liang H, Miao M, et al. In utero beta‐2‐adrenergic agonists exposure and risk of epilepsy: a Danish nationwide population‐based cohort study. *Pharmacoepidemiology and Drug Safety* 2018; 27(11): 1200–1208.

[R16] Chung CP, Dupont WD, Murray KT, Hall K, Stein CM, Ray WA. Comparative out‐of‐hospital mortality of long‐acting opioids prescribed for non‐cancer pain: a retrospective cohort study. *Pharmacoepidemiology and Drug Safety* 2019; 28(1): 48–53.

[R17] Czaja AS, Ross ME, Liu W, et al. Electronic health record (EHR) based postmarketing surveillance of adverse events associated with pediatric off‐label medication use: a case study of short‐acting beta‐2 agonists and arrhythmias. *Pharmacoepidemiology and Drug Safety* 2018; 27(7): 815–822.

[R18] De Landaluce LO, Carbonell P, Asensio C, Escoda N, López P, Laporte JR. Gabapentin and pregabalin and risk of atrial fibrillation in the elderly: a population‐based cohort study in an electronic prescription database. *Drug Safety* 2018; 41(12): 1325–1331.

[R19] Dellay B, Sexter A, Wang JH, Hess GP, Ray Kim W, Israni AK. Impact of sofosbuvir‐based therapy on liver transplant candidates with hepatitis C virus infection. *Pharmacotherapy: The Journal of Human Pharmacology and Drug Therapy* 2019; 39(4): 424–432.

[R20] Dong YH, Chang CH, Wu LC, Hwang JS, Toh S. Comparative cardiovascular safety of nonsteroidal anti‐inflammatory drugs in patients with hypertension: a population‐based cohort study. *British Journal of Clinical Pharmacology* 2018; 84(5): 1045–1056.

[R21] Duong M, Abouelfath A, Lassalle R, Droz C, Blin P, Moore N. Coronary events after dispensing of ibuprofen: a propensity score‐matched cohort study versus paracetamol in the French nationwide claims database sample. *Drug Safety* 2018; 41(11): 1049–1058.

[R22] Eworuke E, Welch EC, Tobenkin A, Maro JC. Use of FDA's Sentinel system to quantify seizure risk immediately following new ranolazine exposure. *Drug Safety* 2019; 42(7): 897–906.

[R23] Frost DA, Soric MM, Kaiser R, Neugebauer RE. Efficacy of tramadol for pain management in patients receiving strong cytochrome P450 2D6 inhibitors. *Pharmacotherapy: The Journal of Human Pharmacology and Drug Therapy* 2019; 39(6): 724–729.

[R24] Gilsenan A, Fortuny J, Cainzos‐Achirica M, et al. Cardiovascular safety of prucalopride in patients with chronic constipation: a multinational population‐based cohort study. *Drug Safety* 2019; 42(10): 1179–1190.

[R25] Gokhale M, Buse JB, DeFilippo Mack C, et al. Calendar time as an instrumental variable in assessing the risk of heart failure with antihyperglycemic drugs. *Pharmacoepidemiology and Drug Safety* 2018; 27(8): 857–866.

[R26] Grzeskowiak LE, Leggett C, Costi L, Roberts CT, Amir LH. Impact of serotonin reuptake inhibitor use on breast milk supply in mothers of preterm infants: a retrospective cohort study. *British Journal of Clinical Pharmacology* 2018; 84(6): 1373–1379.

[R27] Grzeskowiak LE, Amir LH, Smithers LG. Longer‐term breastfeeding outcomes associated with domperidone use for lactation differs according to maternal weight. *European Journal of Clinical Pharmacology* 2018; 74(8): 1071–1075.

[R28] Harding BN, Weiss NS, Walker RL, Larson EB, Dublin S. Proton pump inhibitor use and the risk of fractures among an older adult cohort. *Pharmacoepidemiology and Drug Safety* 2018; 27(6): 596–603.

[R29] Hart LA, Marcum ZA, Gray SL, Walker RL, Crane PK, Larson EB. The association between central nervous system‐active medication use and fall‐related injury in community‐dwelling older adults with dementia. *Pharmacotherapy: The Journal of Human Pharmacology and Drug Therapy* 2019; 39(5): 530–543.

[R30] Hart E, Dunn TE, Feuerstein S, Jacobs DM. Proton pump inhibitors and risk of acute and chronic kidney disease: a retrospective cohort study. *Pharmacotherapy: The Journal of Human Pharmacology and Drug Therapy* 2019; 39(4): 443–453.

[R31] Hawn JM, Wanek M, Bauer SR, et al. Effectiveness, safety, and economic comparison of two inhaled epoprostentol products (Flolan and Veletri) in cardiothoracic surgery patients. *Annals of Pharmacotherapy* 2018; 52(10): 956–964.

[R32] Hellfritzsch M, Rasmussen L, Hallas J, Pottegård A. Using the symmetry analysis design to screen for adverse effects of non‐vitamin K antagonist oral anticoagulants. *Drug Safety* 2018; 41(7): 685–695.

[R33] Hou WH, Chang KC, Li CY, Ou HT. Dipeptidyl peptidase‐4 inhibitor use is associated with decreased risk of fracture in patients with type 2 diabetes: a population‐based cohort study. *British Journal of Clinical Pharmacology* 2018; 84(9): 2029–2039.

[R34] Howard ML, Hossaini R, Tolar C, Gaviola ML. Efficacy and safety of appetite‐stimulating medications in the inpatient setting. *Annals of Pharmacotherapy* 2019; 53(3): 261–267.

[R35] Imatoh T, Nishi T, Yasui M, et al. Association between dipeptidyl peptidase‐4 inhibitors and urinary tract infection in elderly patients: A retrospective cohort study. *Pharmacoepidemiology and Drug Safety* 2018; 27(8): 931–939.

[R36] Iwagami M, Tomlinson LA, Mansfield KE, Douglas IJ, Smeeth L, Nitsch D. Gastrointestinal bleeding risk of selective serotonin reuptake inhibitors by level of kidney function: a population‐based cohort study. *British Journal of Clinical Pharmacology* 2018; 84(9): 2142–2151.

[R37] Janssen PW, Bergmeijer TO, Vos GJA, et al. Tailored P2Y 12 inhibitor treatment in patients undergoing non‐urgent PCI—the POPular Risk Score study. *European Journal of Clinical Pharmacology* 2019; 75(9): 1201–1210.

[R38] Joshi K, Boettiger D, Kerr S, et al. Changes in renal function with long‐term exposure to antiretroviral therapy in HIV infected adults in Asia. *Pharmacoepidemiology and Drug Safety* 2018; 27(11): 1209–1216.

[R39] Jung M, Lee S. Effects of statin therapy on the risk of intracerebral hemorrhage in Korean patients with hyperlipidemia. *Pharmacotherapy: The Journal of Human Pharmacology and Drug Therapy* 2019; 39(2): 129–139.

[R40] Kaplan S, Goehring Jr EL, Melamed‐Gal S, Nguyen‐Khoa BA, Knebel H, Jones JK. Modafinil and the risk of cardiovascular events: findings from three US claims databases. *Pharmacoepidemiology and Drug Safety* 2018; 27(11): 1182–1190.

[R41] Karp I, Sivaswamy A, Booth C. Does the use of incretin‐based medications increase the risk of cancer in patients with type‐2 diabetes mellitus? *Pharmacoepidemiology and Drug Safety* 2019; 28(4): 489–499.

[R42] Kido K, Ngorsuraches S. Comparing the efficacy and safety of direct oral anticoagulants with warfarin in the morbidly obese population with atrial fibrillation. *Annals of Pharmacotherapy* 2019; 53(2): 165–170.

[R43] Kim HA, Lee JY, Park SH, Kang J, Choi KS, Rhie SJ. Clinical outcomes and risk factors of thromboprophylaxis with rivaroxaban versus aspirin in patients undergoing hip arthroplasty in low‐incidence population: A nationwide study in Korea. *Pharmacoepidemiology and Drug Safety* 2019; 28(4): 507–514.

[R44] Klil‐Drori AJ, Santella C, Tascilar K, Yin H, Aprikian A, Azoulay L. Androgen deprivation therapy for prostate cancer and the risk of rheumatoid arthritis: a population‐based cohort study. *Drug Safety* 2019; 42(8): 1005–1011.

[R45] Korol S, White M, O'Meara E, et al. Is there a potential association between spironolactone and the risk of new‐onset diabetes in a cohort of older patients with heart failure? *European Journal of Clinical Pharmacology* 2019; 75(6): 837–847.

[R46] Lai ECC, Shin JY, Kubota K, et al. Comparative safety of NSAIDs for gastrointestinal events in Asia‐Pacific populations: a multi‐database, international cohort study. *Pharmacoepidemiology and Drug Safety* 2018; 27(11): 1223–1230.

[R47] Lee J, Lee S. Comparative effectiveness of combination therapy with statins and angiotensin‐converting enzyme inhibitors versus angiotensin II receptor blockers in patients with coronary heart disease: a nationwide population‐based cohort study in Korea. *Pharmacotherapy: The Journal of Human Pharmacology and Drug Therapy* 2018; 38(11): 1095–1105.

[R48] Lee S, Morris A, Kim S, Li F, Baumgartner L. Impact of quetiapine therapy on QTc prolongation in critically ill patients. *Annals of Pharmacotherapy* 2019; 53(7): 705–710.

[R49] Lin J, Han Z, Wang C, et al. Dual therapy with clopidogrel and aspirin prevents early neurological deterioration in ischemic stroke patients carrying CYP2C19* 2 reduced‐function alleles. *European Journal of Clinical Pharmacology* 2018; 74(9): 1131–1140.

[R50] Lin HW, Ho YF, Lin FJ. Statin use associated with lower risk of epilepsy after intracranial hemorrhage: a population‐based cohort study. *British Journal of Clinical Pharmacology* 2018; 84(9): 1970–1979.

[R51] Liu W, Antonelli PJ, Dahm P, et al. Risk of sudden sensorineural hearing loss in adults using phosphodiesterase type 5 inhibitors: Population‐based cohort study. *Pharmacoepidemiology and Drug Safety* 2018; 27(6): 587–595.

[R52] MacDonald SC, McElrath TF, Hernández‐Díaz S. Use and safety of disease‐modifying therapy in pregnant women with multiple sclerosis. *Pharmacoepidemiology and Drug Safety* 2019; 28(4): 556–560.

[R53] Makris UE, Alvarez CA, Mortensen EM, Mansi IA. Association of statin use with increased risk of musculoskeletal conditions: a retrospective cohort study. *Drug Safety* 2018; 41(10): 939–950.

[R54] Malfertheiner P, Ripellino C, Cataldo N. Severe intestinal malabsorption associated with ACE inhibitor or angiotensin receptor blocker treatment. An observational cohort study in Germany and Italy. *Pharmacoepidemiology and Drug Safety* 2018; 27(6): 581–586.

[R55] Martinez BK, Baker WL, Sood NA, et al. Influence of polypharmacy on the effectiveness and safety of rivaroxaban versus warfarin in patients with nonvalvular atrial fibrillation. *Pharmacotherapy: The Journal of Human Pharmacology and Drug Therapy* 2019; 39(2): 196–203.

[R56] Maura G, Billionnet C, Coste J, Weill A, Neumann A, Pariente A. Non‐bleeding adverse events with the use of direct oral anticoagulants: a sequence symmetry analysis. *Drug Safety* 2018; 41(9): 881–897.

[R57] McCrory BE, Harper HN, McPhail GL. Use and incidence of adverse effects of proton pump inhibitors in patients with cystic fibrosis. *Pharmacotherapy: The Journal of Human Pharmacology and Drug Therapy* 2018; 38(7): 725–729.

[R58] Meade LT, Mannka ML. The effect of glucagon‐like peptide‐1 receptor agonists and sodium‐glucose cotransporter‐2 inhibitors in patients prescribed regular U‐500 insulin. *Annals of Pharmacotherapy* 2019; 53(11): 1111–1116.

[R59] Mesrine S, Gusto G, Clavel‐Chapelon F, Boutron‐Ruault MC, Fournier A. Use of benzodiazepines and cardiovascular mortality in a cohort of women aged over 50 years. *European Journal of Clinical Pharmacology* 2018; 74(11): 1475–1484.

[R60] Mueller T, Alvarez‐Madrazo S, Robertson C, Wu O, Bennie M. Comparative safety and effectiveness of direct oral anticoagulants in patients with atrial fibrillation in clinical practice in Scotland. *British Journal of Clinical Pharmacology* 2019; 85(2): 422–431.

[R61] Mugusi S, Ngaimisi E, Janabi M, et al. Neuropsychiatric manifestations among HIV‐1 infected African patients receiving efavirenz‐based cART with or without tuberculosis treatment containing rifampicin. *European Journal of Clinical Pharmacology* 2018; 74(11): 1405–1415.

[R62] Navarro J, Santos JR, Silva A, et al. Effectiveness of once/day dolutegravir plus boosted darunavir as a switch strategy in heavily treated patients with human immunodeficiency virus. *Pharmacotherapy: The Journal of Human Pharmacology and Drug Therapy* 2019; 39(4): 501–507.

[R63] O'Connell M, Sandgren M, Frantzen L, Bower E, Erickson B. Medical cannabis: effects on opioid and benzodiazepine requirements for pain control. *Annals of Pharmacotherapy* 2019; 53(11): 1081–1086.

[R64] Oshagbemi OA, Franssen FM, Braeken DC, et al. Blood eosinophilia, use of inhaled corticosteroids, and risk of COPD exacerbations and mortality. *Pharmacoepidemiology and Drug Safety* 2018; 27(11): 1191–1199.

[R65] Park SK, Baek YH, Pratt N, Ellett LK, Shin JY. The uncertainty of the association between proton pump inhibitor use and the risk of dementia: prescription sequence symmetry analysis using a Korean healthcare database between 2002 and 2013. *Drug Safety* 2018; 41(6): 615–624.

[R66] Pearce JA, Shiltz DL, Ding Q. Effectiveness and safety comparison for systemic corticosteroid therapy with and without inhaled corticosteroids for COPD exacerbation management. *Annals of Pharmacotherapy* 2018; 52(11): 1070–1077.

[R67] Policardo L, Seghieri G, Gualdani E, Franconi F. Effect of statins in preventing hospitalizations for infections: a population study. *Pharmacoepidemiology and Drug Safety* 2018; 27(8): 878–884.

[R68] Powell MZ, Mueller SW, Reynolds PM. Assessment of opioid cross‐reactivity and provider perceptions in hospitalized patients with reported opioid allergies. *Annals of Pharmacotherapy* 2019; 53(11): 1117–1123.

[R69] Quinn CS, Jorgenson MR, Descourouez JL, Muth BL, Astor BC, Mandelbrot DA. Management of tumor necrosis factor *α* inhibitor therapy after renal transplantation: a comparative analysis and associated outcomes. *Annals of Pharmacotherapy* 2019; 53(3): 268–275.

[R70] Sahloff EG, Duggan JM. Clinical outcomes associated with once‐daily ritonavir‐boosted darunavir plus tenofovir/emtricitabine in HIV‐infected patients harboring at minimum a M184V/I resistance mutation. *Annals of Pharmacotherapy* 2019; 53(1): 50–55.

[R71] Schafer JH, Casey AL, Dupre KA, Staubes BA. Safety and efficacy of apixaban versus warfarin in patients with advanced chronic kidney disease. *Annals of Pharmacotherapy* 2018; 52(11): 1078–1084.

[R72] Seong JM, Yee J, Gwak HS. Dipeptidyl peptidase‐4 inhibitors lower the risk of autoimmune disease in patients with type 2 diabetes mellitus: a nationwide population‐based cohort study. *British Journal of Clinical Pharmacology* 2019; 85(8): 1719–1727.

[R73] Sheu JJ, Tsai MT, Erickson SR, Wu CH. Association between anticholinergic medication use and risk of dementia among patients with Parkinson's disease. *Pharmacotherapy: The Journal of Human Pharmacology and Drug Therapy* 2019; 39(8): 798–808.

[R74] Simeonova M, Vries dF, Pouwels S, et al. Increased risk of all‐cause mortality associated with domperidone use in Parkinson's patients: a population‐based cohort study in the UK. *British Journal of Clinical Pharmacology* 2018; 84(11): 2551–2561.

[R75] Siodlak M, Jorgenson MR, Descourouez JL, et al. Impact of high‐dose acyclovir cytomegalovirus prophylaxis failure in abdominal solid organ transplant recipients. *Pharmacotherapy: The Journal of Human Pharmacology and Drug Therapy* 2018; 38(7): 694–700.

[R76] Spence AD, Busby J, Hughes CM, Johnston BT, Coleman HG, Cardwell CR. Statin use and survival in patients with gastric cancer in two independent population‐based cohorts. *Pharmacoepidemiology and Drug Safety* 2019; 28(4): 460– 470.

[R77] Spoendlin J, Gagne JJ, Lewey JJ, Patorno E, Schneeweiss S, Desai RJ. Comparative effectiveness and safety of antiplatelet drugs in patients with diabetes mellitus and acute coronary syndrome. *Pharmacoepidemiology and Drug Safety* 2018; 27(12): 1361–1370.

[R78] Squires PJ, Pahor M, Manini TM, Brown JD. Effect of Gastric Acid Suppressants on Response to a Physical Activity Intervention and Major Mobility Disability in Older Adults: Results from the Lifestyle Interventions for Elders (LIFE) Study. *Pharmacotherapy: The Journal of Human Pharmacology and Drug Therapy* 2019; 39(8): 816–826.

[R79] Svanström H, Lund M, Melbye M, Pasternak B. Concomitant use of low‐dose methotrexate and NSAIDs and the risk of serious adverse events among patients with rheumatoid arthritis. *Pharmacoepidemiology and Drug Safety* 2018; 27(8): 885–893.

[R80] Svanström H, Lund M, Melbye M, Pasternak B. Use of proton pump inhibitors and the risk of acute kidney injury among patients with rheumatoid arthritis: cohort study. *Drug Safety* 2018; 41(8): 817–826.

[R81] Trivedi LU, Alvarez CA, Mansi IA. Association of statin therapy with risk of epilepsy in 2 propensity score–matched cohorts. *Annals of Pharmacotherapy* 2018; 52(6): 546–553.

[R82] Tsai SC, Sheu SY, Chien LN, Lee HC, Yuan EJS, Yuan RY. High exposure compared with standard exposure to metoclopramide associated with a higher risk of parkinsonism: a nationwide population‐based cohort study. *British Journal of Clinical Pharmacology* 2018; 84(9): 2000–2009.

[R83] Ujeyl M, Köster I, Wille H, et al. Comparative risks of bleeding, ischemic stroke and mortality with direct oral anticoagulants versus phenprocoumon in patients with atrial fibrillation. *European Journal of Clinical Pharmacology* 2018; 74(10): 1317–1325.

[R84] Walsh CA, Cahir C, Bennett KE. Association between adherence to antihypertensive medications and health outcomes in middle and older aged community dwelling adults; results from the Irish longitudinal study on aging. *European Journal of Clinical Pharmacology* 2019; 75(9): 1283–1292.

[R85] Wang IK, Lin CL, Yen TH, Lin SY, Yao‐Lung L, Sung FC. Icodextrin reduces the risk of congestive heart failure in peritoneal dialysis patients. *Pharmacoepidemiology and Drug Safety* 2018; 27(4): 447–452.

[R86] Wang CY, Fu SH, Yang RS, Hsiao FY. Use of dipeptidyl peptidase‐4 inhibitors and the risk of arthralgia: population‐based cohort and nested case–control studies. *Pharmacoepidemiology and Drug Safety* 2019; 28(4): 500–506.

[R87] Winkel JS, Damkier P, Hallas J, Henriksen DP. Treatment with montelukast and antidepressive medication—a symmetry analysis. *Pharmacoepidemiology and Drug Safety* 2018; 27(12): 1409–1415.

[R88] Young JC, Lund JL, Dasgupta N, Jonsson Funk M. Opioid tolerance and clinically recognized opioid poisoning among patients prescribed extended‐release long‐acting opioids. *Pharmacoepidemiology and Drug Safety* 2019; 28(1): 39–47.

[R89] Zielinski GD, Teichert M, Klok FA, et al. Direct oral anticoagulant use and subsequent start of proton pump inhibitors as proxy for gastric complaints. *Pharmacoepidemiology and Drug Safety* 2018; 27(12): 1371–1378.
