# Supplementary material for: New‐user and prevalent‐user designs and the definition of study time origin in pharmacoepidemiology: A review of reporting practices
Source: Pharmacoepidemiol Drug Saf. 2021 May 10;30(7):960–74. doi: 10.1002/pds.5258 (PMC8252086; doi:10.1002/pds.5258)
Supplement: Supplementary file 1 — Data S1. [file PDS-30-960-s001.pdf]

SUPPLEMENTARY FILE

This Supplementary File accompanies the manuscript "New-user and prevalent-user designs and the definition of study time origin in pharmacoepidemiology: a review of reporting practices" and contains the search string for the systematic review as well as the background on defining a study time origin.

SEARCH STRING

We performed a PubMed search on February 3rd 2020 which returned 2, 457 records.

(("Pharmacoepidemiology AND drug safety"[Journal]) OR "Pharmacotherapy"[Journal] OR "British journal of clinical pharmacology"[Journal] OR "The Annals of pharmacotherapy"[Journal] OR "Drug safety"[Journal] OR "European journal of clinical pharmacology"[Journal]) AND ("2016/01/01"[Date - Entrez] : "2019/07/01"[Date - Entrez]) NOT (“case reports”[Publication Type] OR “comment”[Publication Type] OR "clinical trial, phase i"[Publication Type] OR "clinical trial, phase ii"[Publication Type] OR "clinical trial, phase iii"[Publication Type] OR “dictionary”[Publication Type] OR “editorial”[Publication Type] OR "historical article"[Publication Type] OR "interview"[Publication Type] OR “lectures”[Publication Type] OR "legislation"[Publication Type] OR "letter"[Publication Type] OR "meta analysis"[Publication Type] OR "personal narratives"[Publication Type] OR "randomized controlled trial"[Publication Type] OR "review"[Publication Type])

BACKGROUND ON DEFINING A STUDY TIME ORIGIN

Here, we briefly review common biases that can be introduced by inappropriate designation of follow-up time in observational studies of drug effects (for a more elaborate discussion, see e.g. [1,2,3,4,5,6,7,8,9]). Ideally, a study is designed such that operational decisions match the specified causal contrast of interest, i.e., the target causal effect or so-called estimand. The target causal effect specifies a causal contrast of treatment strategies for a particular target population, which determines the choice for the comparison made in a study, i.e., the study exposure arm and (active) comparator arm in a study. Whether the target causal effect can be estimated from the available data depends on whether it is convincing that untestable identifying assumptions hold, i.e., conditional exchangeability, positivity, and consistency. The choice of the time origin of a study design is directly linked to the target causal effect because the estimated outcome risk refers to the (cumulative) probability of an event of interest occurring over time since a given origin in a specific population[8]. The concept study time origin refers to the moment from which onwards individuals contribute to a particular study conducted within a cohort (e.g., an electronic health records database). The study time origin can be operationalized relative to the moment when individuals meet eligibility criteria, are assigned to a treatment and follow-up is started as will be illustrated in various examples throughout the current study.

Figure 1a is a schematic depiction of a target causal effect (for example an intention-to-treat or per-protocol effect) of a binary treatment on a certain outcome, where the study exposure arm and comparator arm are assumed to be comparable conditional on measured confounders (i.e., there is conditional exchangeability). An archetypical study design that potentially allows identification of the causal effect of interest from empirical data is the *active-comparator incident-user design*. When an appropriate active comparator is chosen, in the sense that the comparison group reflects a clinically meaningful alternative treatment option in real-world practice, the active-comparator incident-user design increases the likelihood of achieving conditional exchangeability[10].

When prevalent users of treatment are included in a study, the follow-up of those individuals is left truncated and omitted from the analysis (Figure 1b). For permanent outcomes, of which 'death' is arguably the clearest example, individuals included in the analysis did not develop the outcome during the exposed period before start of follow-up (the cohort is depleted of susceptibles). Consequently, including prevalent users can lead to under-ascertainment of events early in the course of treatment and increases the risk of controlling for confounding factors that were affected by the treatment[11,6]. Since much information is unobserved, it will be difficult to specify a set of covariates that is sufficient to achieve conditional exchangeability of treatment groups. In many cases, it is unlikely that a prevalent-user study design is suitable to identify the specified causal effect of interest. Moreover, a target causal effect cannot be clearly defined for individuals who have been using a treatment for an unknown period of time (prevalent users).

Another issue is that individuals could be assigned to a treatment group based on the treatment strategy observed after start of follow-up, rather than the treatment strategy at the time of start of follow-up[5] (Figure 1c), for example when individuals are classified as 'users' only after they filled a number (e.g., three) of prescriptions of that treatment. In that case, individuals cannot experience the outcome during the first three prescriptions. This period is often referred to as immortal time. When some individuals are immortal for part of the time they were followed-up, it seems unlikely that the causal effect of interest can be identified. A possible solution would be to reset the start of follow-up until after the eligibility criterion has been met, i.e., when the number of required prescriptions has been filled. Notably, this should be done for both treatment arms to create interpretable effects, like "what would the difference in outcomes for these treatments be if everyone filled at least three prescriptions and death could be prevented during the time until they filled a third prescription?".

A final issue is time-lag bias, which occurs when follow-up is started at the moment of treatment initiation, but the compared treatments are prescribed at different stages of the disease[12]. For instance, if the effect of a first-line drug is compared to a second-line drug and the start of follow-up for both the active and comparator arm is defined by treatment initiation, the disease stage differs between the treatment groups. As capturing this difference in disease progress in measured covariates is hardly feasible[13], time-lag bias likely jeopardizes the (conditional) exchangeability of treatment groups. When protocol adherence to switch to second-line treatment is strict, the incomparability of treatment groups may be so extreme that non-positivity is introduced. Interestingly, the moment of meeting eligibility criteria, treatment initiation and start of follow-up may be aligned in situations where time-lag bias occurs (Figure 1d), but that results in a comparison of groups at different stages of the disease. As a possible solution, the start of follow-up for the first-line drug could be reset to the time since diagnosis that is comparable to the time since diagnosis at which follow-up for the second-line drug started, where exchangeability conditional on covariates should still be considered.

An incident user can more generally be defined as a new user of any treatment decision, i.e., initiating a treatment, but also switching to a different treatment or a change of dose. All of the examples above related to initiation of treatment can be translated to different treatment decisions by evaluating the plausibility of identifiability assumptions in light of the particular treatment decision. This understanding of the incident-user design was introduced by Brookhart[14], expanded to prevalent new-users of treatment by Suissa[15].

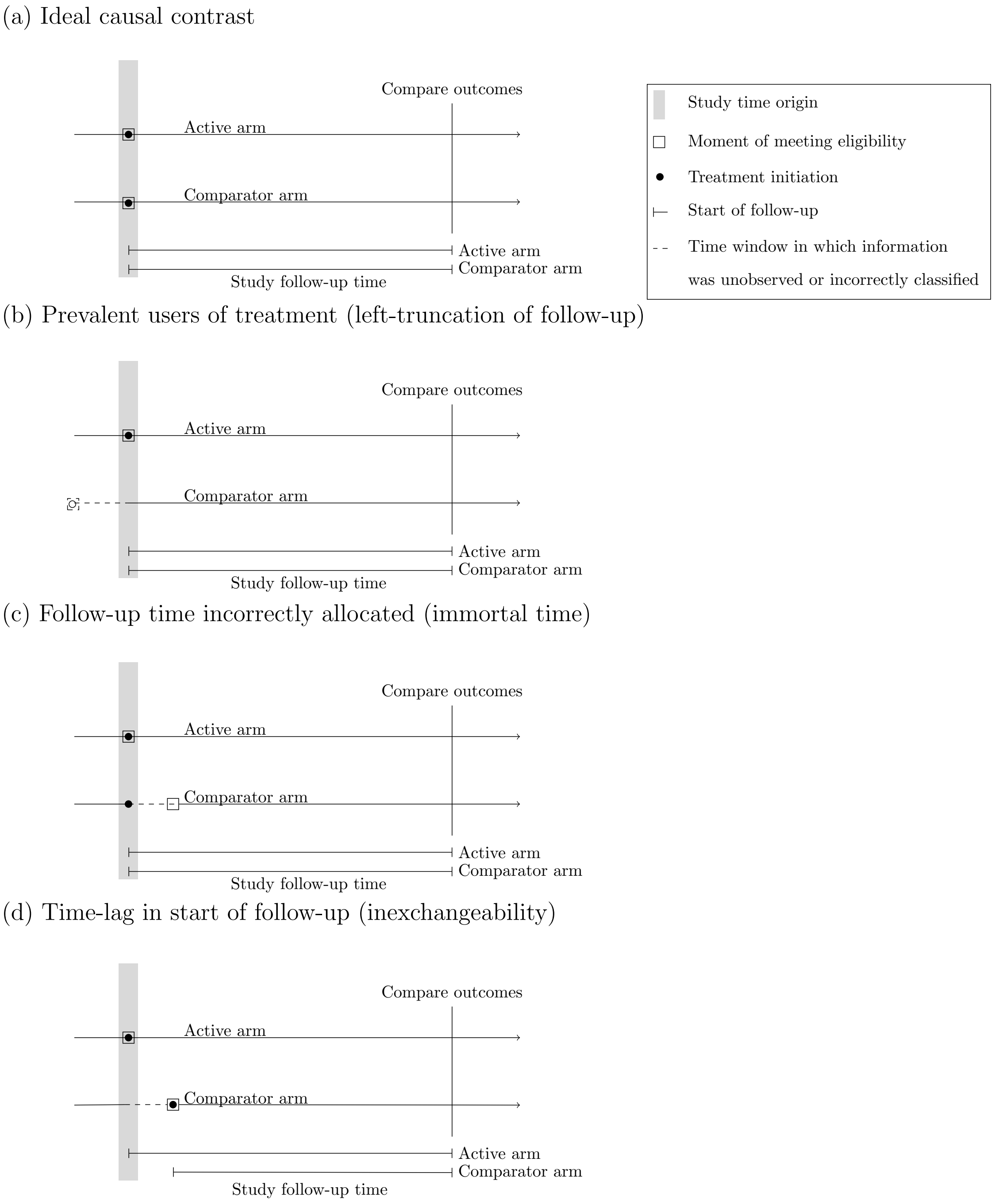

FIGURE 1 Subfigure (a) depicts a simplified ideal causal contrast for a binary treatment. Subfigures (b) - (d) depict possible biases that can be introduced by inappropriate designation of follow-up time. In empirical studies, the specified design flaws can occur in either treatment arm, both treatment arms, and combination of different flaws can take place. For simplification, we presented a single design flaw in the comparator treatment arm in each subfigure (b) - (d). Study design flaws may lead to violation of identifying assumptions, as is explained in Section 2 of the main text. The dotted lines in the figure thus indicate the consideration whether exchangeability of treatment groups is jeopardized by the misalignment and whether this can be corrected by measured covariates, or non-positivity is introduced.

References

1. Maringe C, Benitez Majano S, Exarchakou A, et al. Reflections on modern methods: trial emulation in the presence of immortal-time bias. Assessing the benefit of major surgery for elderly lung cancer patients using observational data. *International Journal of Epidemiology* 2020.

2. Platt R, Hutcheon J, Suissa S. Immortal Time Bias in Epidemiology. *Current Epidemiology Reports* 2019; 6(1): 23–27.

3. Lund JL, Richardson DB, Stürmer T. The active comparator, new user study design in pharmacoepidemiology: historical foundations and contemporary application. *Current epidemiology reports* 2015; 2(4): 221–228.

4. Johnson ES, Bartman BA, Briesacher BA, et al. The incident user design in comparative effectiveness research. *Pharmacoepidemiology and drug safety* 2013; 22(1): 1–6.

5. Suissa S. Immortal time bias in pharmacoepidemiology. *American journal of epidemiology* 2007; 167(4): 492–499.

6. Ray WA. Evaluating medication effects outside of clinical trials: new-user designs. *American journal of epidemiology* 2003; 158(9): 915–920.

7. Hernán MA, Sauer BC, Hernández-Díaz S, Platt R, Shrier I. Specifying a target trial prevents immortal time bias and other self-inflicted injuries in observational analyses. *Journal of clinical epidemiology* 2016; 79: 70–75.

8. Edwards JK, Hester LL, Gokhale M, Lesko CR. Methodologic issues when estimating risks in pharmacoepidemiology. *Current epidemiology reports* 2016; 3(4): 285–296.

9. Farewell V, Cox D. A note on multiple time scales in life testing. *Journal of the Royal Statistical Society: Series C (Applied Statistics)* 1979; 28(1): 73–75.

10. Velentgas P, Dreyer NA, Nourjah P, Smith SR, Torchia MM, others . *Developing a protocol for observational comparative effectiveness research: a user's guide*. Government Printing Office . 2013.

11. Hernán MA. Counterpoint: epidemiology to guide decision-making: moving away from practice-free research. *American journal of epidemiology* 2015; 182(10): 834–839.

12. Suissa S, Azoulay L. Metformin and the risk of cancer: time-related biases in observational studies. *Diabetes care* 2012; 35(12): 2665–2673.

13. Bosco JL, Silliman RA, Thwin SS, et al. A most stubborn bias: no adjustment method fully resolves confounding by indication in observational studies. *Journal of clinical epidemiology* 2010; 63(1): 64–74.

14. Brookhart MA. Counterpoint: the treatment decision design. *American journal of epidemiology* 2015; 182(10): 840–845.

15. Suissa S, Moodie EE, Dell'Aniello S. Prevalent new-user cohort designs for comparative drug effect studies by time-conditional propensity scores. *Pharmacoepidemiology and drug safety* 2017; 26(4): 459–468.

□
